# Phage endolysins as alternative antimicrobials: mechanisms, clinical progress, and emerging resistance frameworks

**DOI:** 10.3389/fmicb.2026.1762768

**Published:** 2026-03-24

**Authors:** Haocong Qian, Yuxuan Zheng

**Affiliations:** 1 School of Life Sciences, Fudan University, Shanghai, China; 2 Human Phenome Institute, Pudong Hosptial, Fudan University, Shanghai, China

**Keywords:** antibiotic alternatives, antimicrobial resistance, phage endolysin resistance, phage endolysins, phage therapy

## Abstract

Phage endolysins are increasingly recognized as alternatives to antibiotics for mitigating the growing threat of antimicrobial resistance. Since their initial identification in the 1950s, phage endolysins have been extensively demonstrated to effectively combat bacterial infections in animal models and human patients. Although phage endolysins have completed Phase II and III clinical trials, potential obstacles and challenges associated with their large-scale use remain largely undefined. This review examines the potential of phage endolysins and the challenges they face in addressing infections caused by antibiotic-resistant bacteria in the future. We conducted a comprehensive overview of the historical development, bactericidal mechanisms, clinical progress, and resistance of phage endolysins. The information presented herein may facilitate the design of novel, potent endolysins and provide strategic insights for addressing phage endolysin resistance.

## 1 Introduction

Antimicrobial resistance (AMR) is a significant global health challenge in the 21st century. Predictions indicate that by 2050, AMR will be responsible for approximately 1.91 million deaths annually worldwide, along with an additional 8.22 million deaths each year due to AMR-related diseases. Cumulatively, from 2025 to 2050, an estimated 39.1 million individuals are expected to die as a result of AMR (GBD 2021 Antimicrobial Resistance Collaborators, 2024). Phage lysins include endolysins and virion-associated peptidoglycan hydrolases (VAPGHs; Gutiérrez et al., 2018). Phage endolysins typically consist of one or two N-terminal catalytic domains and a C-terminal cell wall binding domain (CBD). Differently, VAPGH contains one or two catalytic domains but lacks a known CBD (Rodríguez-Rubio et al., 2013). Endolysins, a phage-encoded enzyme produced by a double-stranded DNA phage, works synergistically with holin to hydrolyze the bacterial host cell wall before phage egress. Canonical holins form very large holes that allow nonspecific release of fully-folded proteins, another class of holins, the pinholins, make much smaller holes, or pinholes, that serve only to depolarize the membrane (Woźnica et al., 2015; Pang et al., 2009). It is considered a promising candidate for antibiotic alternatives (Wang et al., 2000). The first research demonstrating that phages encode proteins capable of lysing bacteria, known as phage endolysins, was conducted in Jacob and Fuerst (1958). In fact, researchers had already discovered signs of the existence of lysins from phage before 1958 (Bronfenbrenner et al., 1925; Ralston et al., 1957). In the following year, group C streptococci phage-associated lysin was purified for the first time, proving its ability to lyse Group A streptococci and the peptidoglycan of Group A streptococci (Freimer et al., 1959). In 1987, the structure of the endolysin from phage T4 was revealed for the first time (Weaver and Matthews, 1987). The initial structural elucidation of phage endolysin provides researchers with valuable insights into its mechanism of action. In Díaz et al. (1990), pioneered the construction of chimeric endolysins and investigated the underlying mechanisms of action within endolysins domains. Phage endolysin CF-301, CF-296, SAL-200 have undergone clinical trials and demonstrated significant potential for the treatment of bacterial infections (Cassino et al., 2016; Karau et al., 2022; Jun et al., 2017; Jun et al., 2016; Jun et al., 2014; Fowler et al., 2024). And phage endolysins have emerged over the past two decades as a green and highly effective alternative to conventional antibiotics. However, certain endolysins may present limitations, such as a relatively short *in vivo* half-life and the potential to induce inflammatory cytokines as well as neutralizing antibodies (Jado et al., 2003; Nau and Eiffert, 2002). Extensive research has elucidated the modes of action of phage endolysins, explored the application of native endolysins, engineered enzyme variants with enhanced properties, and developed nanocarrier-based delivery systems. These advances aimed to address challenges in public health, agriculture, food safety, animal disease control, and related fields (Nelson et al., 2001). Meanwhile, our current understanding of resistance to phage endolysins remains limited, and the potential risks associated with their long-term and extensive use have yet to be fully elucidated. In this review, we provided a comprehensive overview of the historical development and bactericidal mechanisms of phage lysins. Subsequently, we discussed the advances in their application in human and animal settings. Finally, to address the potential crisis of bacterial resistance to the future application of phage lysins, we explored the possibilities and molecular mechanisms associated with the emergence of phage lysin resistance.

## 2 The mechanism of action of phage endolysins

Most bacteria possess a protective peptidoglycan (PG) in their cell walls. This layer helps them manage internal turgor pressure and protect against external threats (Alvarez et al., 2024). Additionally, PG is a target for phage lysins, which can lyse bacteria. Hence, understanding the structure of PG is essential for comprehending how endolysins kill bacteria. Endolysins are classified based on the specific target sites within the PG structure, with at least five distinct positions in the murein that have been either experimentally confirmed or hypothesized to be cleaved (Payne and Hatfull, 2012; Hermoso et al., 2007). This section summarizes the ways in which phage endolysins lyse Gram-positive and Gram-negative bacteria.

### 2.1 Gram-positive bacteria of phage endolysins

**Figure 1:**
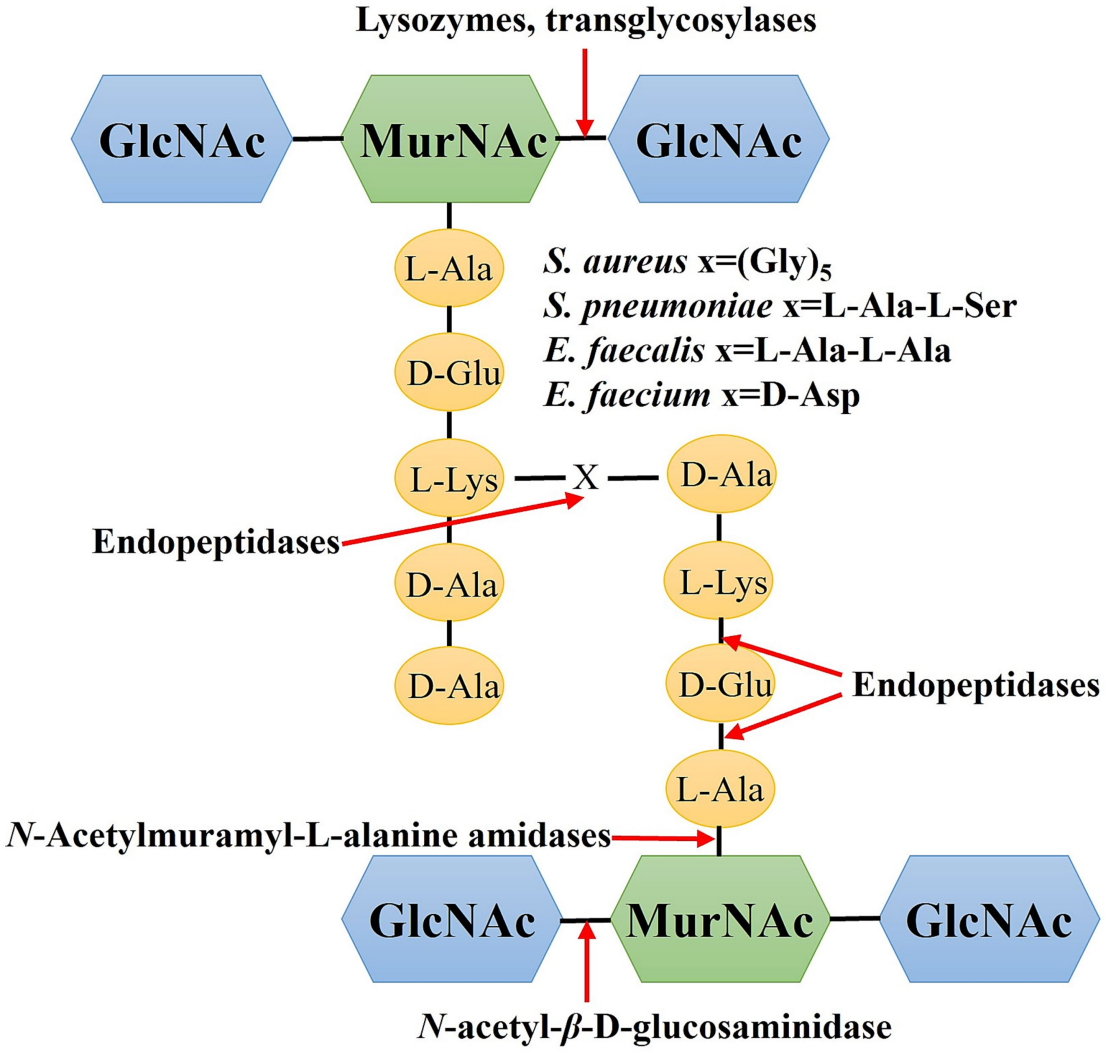
Structural features and enzymatic activities of phage endolysins targeting bacterial peptidoglycan. Phage endolysins are capable of cleaving five specific sites and are classified into five categories: *N*-acetylmuramyl-L-alanine amidase, endopeptidases, lysozymes, transglycosylases, and *N*-acetyl-β-D-glucosaminidase.

To better understand the bactericidal mechanism of phage endolysins, we first need to summarize the structure of endolysin. Phage endolysins that target Gram-positive and Gram-negative bacteria have distinct architectures that reflect the differences in cell wall composition between these two major bacterial groups. Most phage endolysins derived from Gram-positive bacteria contain either one or two catalytic domains (CD) at the N-terminus and a CBD at the C-terminus, with CD and CBD connected by a short peptide linker (Figure 1). Phage endolysins can hydrolyze the essential peptidoglycan linkages in susceptible Gram-positive bacteria, leading to rapid cell lysis (Nelson et al., 2001). Additionally, these endolysins typically have molecular weights ranging from 25 to 40 kDa (Fischetti et al., 2006). CBD specifically binds to the cell wall, positioning the CD for effective contact and cleavage of peptidoglycan (Broendum et al., 2018). CBD also imparts specificity to endolysins by detecting and attaching to ligand molecules embedded in the bacterial cell wall. CBDs with similar structures and conserved sequences contribute to the broad host range of phage endolysins (Schmelcher et al., 2012). Based on the published structure of phage endolysins, the proteins can be broadly classified into four groups: CW_binding_1, SH3b domains, CW_7/PG_binding_1, and *α*/*β* structures. Meanwhile, a novel type of CBD was identified in the endolysin of phage SA97 (LysSA97), which shares a mere 19% homology with other staphylococcal endolysins deposited in the database (Chang and Ryu, 2017). Indeed, there remain numerous undescribed variants among the currently identified CBD types. The previous study has demonstrated the use of repetitive structural motifs as scaffolds to interact with the cell wall, but the mechanism by which the CBD structure expands the host range of lytic enzymes remains unclear (Broendum et al., 2018). SH3b domains have been shown to bind to the peptidoglycan peptide cross-bridge (Gründling and Schneewind, 2006). However, the specific site where some binding domains attaches to the cell wall remains unknown. Consequently, a comprehensive analysis of the structural principles and cell wall binding sites of the CBD will support the rational design of next-generation phage endolysins, particularly when combined with artificial intelligence-driven approaches.

The enzymatic domain responsible for cleaving specific bonds in bacterial peptidoglycan is known as CD. PG of *Staphylococcus aureus* has been extensively studied, and numerous research efforts have explored the use of endolysins to control *S. aureus* infections (Fowler et al., 2024; de Jonge et al., 1992). The cell wall is composed of PG, wall teichoic acids (WTAs), lipoteichoic acids, and surface proteins (Sutton et al., 2021). Generally, the thickness of the cell wall ranges from 20 to 40 nm (Matias and Beveridge, 2007). Given that phage endolysins target peptidoglycan, it is hypothesized that bacteria harboring a thicker peptidoglycan layer might display enhanced resistance to these endolysins. PG is the principal component of the bacterial cell wall, surrounding the cytoplasmic membrane and forming a continuous macromolecule known as the sacculus (Turner et al., 2014). It consists of long glycan strands made up of repeating disaccharide units (*N*-acetylglucosamine [GlcNAc] and *N*-acetylmuramic acid [MurNAc]), which are linked together by *β*-1,4 glycosidic bonds (de Pedro and Cava, 2015). The composition of stem peptides and their cross-linked peptide bridges differ among various Gram-positive bacteria. In *S. aureus*, the D-lactoyl group of each MurNAc residue is replaced by a peptide stem composed of L-Ala-D-Glu-L-Lys-D-Ala. The cross-linking of peptidoglycan strands is facilitated by the formation of a covalent bond between the *α*-carboxyl group of D-alanine at the fourth position and the *ε*-amino group of the meso-diaminopimelic acid (m-DAP) at the third position. This linkage is mediated by a short peptide bridge comprising five glycine residues (Gly₅), although in certain bacterial strains, serine residues may also be incorporated into this bridge (Figure 1; de Pedro and Cava, 2015; Vollmer et al., 2008). Conversely, *Streptococcus pneumoniae* displays a heterogeneous composition, consisting of a mixture of both dipeptide-substituted and unsubstituted stem peptides. The peptide bridge is formed by L-Ala-L-Ala in *Enterococcus faecalis* and D-Asp in *Enterococcus faecium*, respectively (Figure 1; Rajagopal and Walker, 2017).

Based on the structure of PG, phage endolysins are capable of cleaving five specific sites. Specifically, *N*-Acetylmuramyl-L-alanine amidases cleave the amide bond linking MurNAc to L-alanine, effectively separating the glycan strand from the peptide. Endopeptidases cleave a short peptide bridge composed of five Gly residues. Lysozymes and transglycosylases (LTs) cleave the *β*-1,4 glycosidic bonds. The latter cleaves the Mur*N*Ac-Glc*N*Ac glycosidic bond, concurrently generating a 1,6-anhydro ring at Mur*N*Ac via intramolecular transglycosylation. Meanwhile, *N*-acetyl-*β*-D-glucosaminidase only cleave the *β*-1,4 glycosidic bond between Glc*N*Ac and Mur*N*Ac residues (Figure 1; Gutiérrez et al., 2018; Vollmer et al., 2008; Höltje, 1995; Vollmer et al., 2008). Reasonably, the location of the CD domain determines the cleavage mode of endolysins on PG. Lysozymes and LTs that target the exposed PG surface are more likely to cleave the PG layer effectively.

### 2.2 Gram-negative bacteria of phage endolysins

On May 17, 2024, the World Health Organization updated its list of drug-resistant bacteria that pose the greatest threat to human health (WHO, 2024). The 2024 update of the Bacterial Priority Pathogens List now includes 15 families of antibiotic-resistant bacteria, classified into critical, high, and medium categories for prioritization. This list serves as a guide for developing new and essential treatments to curb the spread of AMR. The critical priority pathogens, such as Gram-negative bacteria resistant to last-resort antibiotics and *Mycobacterium tuberculosis* resistant to the antibiotic rifampicin, present major global threats due to their high burden, ability to resist treatment, and potential to spread resistance to other bacteria. Gram-negative bacteria have inherent capabilities to develop new resistance mechanisms and can transfer genetic material that enables other bacteria to become drug-resistant as well. Hence, it is imperative to accelerate the development of new antibacterial agents effective against Gram-negative bacteria.

**Figure 2:**
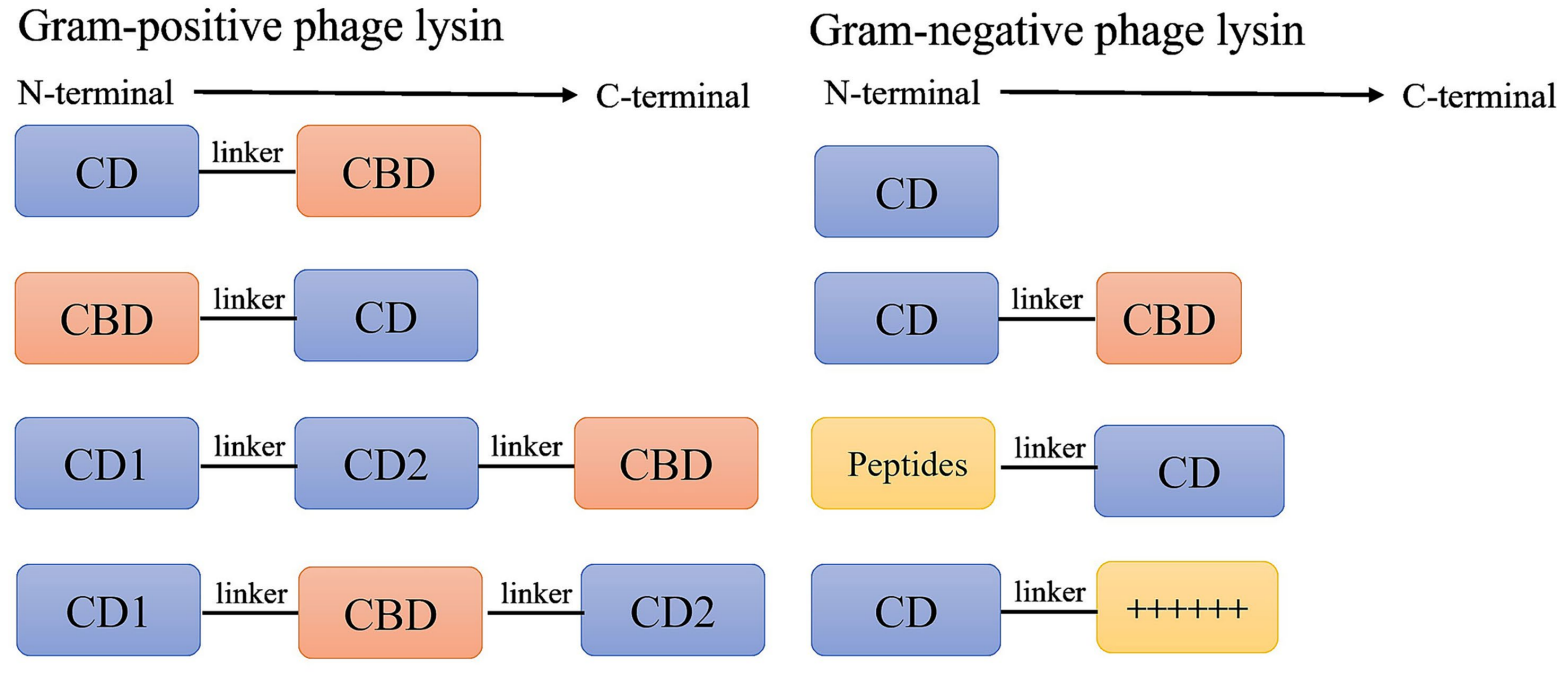
The typical modular structure of phage endolysins for Gram-positive and Gram-negative bacteria. Most phage endolysins derived from Gram-positive bacteria contain either one or two CD and a CBD. Gram-negative endolysins generally consist solely of a CD. Nevertheless, a subset of Gram-negative endolysins incorporates outer-membrane-penetrating peptides alongside a CBD.

The outer membrane (OM) of Gram-negative bacteria acts as a barrier, preventing endolysins from accessing and degrading the underlying peptidoglycan layer. This protection makes Gram-negative bacteria less susceptible to lysin attack (Gondil and Chhibber, 2021). Gram-negative endolysins typically exhibit a globular architecture and contain a single CD that disrupts the cell wall (Schmelcher et al., 2012). These endolysins generally have molecular weights ranging from 15 to 20 kDa and consist solely of a CD (Figure 2). The absence of a CBD in Gram-negative endolysins impedes their capacity for rapid bacterial arrest, thereby diminishing overall lysis efficiency relative to their Gram-positive counterparts (Shah et al., 2023). However, recent advancements, such as endolysins containing OM-penetrating peptides, have enabled effective killing of Gram-negative bacteria. Meanwhile, certain endolysins have been reported to demonstrate limited or context-dependent translocation across the outer membrane under specific conditions (Xu et al., 2004).

Previous studies have demonstrated that endolysins inherently possess the ability to penetrate the OM. *Aeromonas* phage endolysin EndoA3, a Zn^2+^-containing Ca^2+^-dependent l-alanyl-d-glutamate peptidase, exhibits bacteriocidal activity against *Acinetobacter baumannii*, *Escherichia coli*, and *Pseudomonas aeruginosa*, as well as the Gram-positive bacteria of *S. aureus* and *Bacillus subtilis* (Mikoulinskaia et al., 2025). *Pseudomonas phragmitis* lysogenic phage endolysins PlyKp104 possesses an LT domain and exhibits excellent bactericidal activity against *Klebsiella* sp. and *P. aeruginosa* strains. Notably, PlyKp104 reduced all *P. aeruginosa* strains by 3 to 4 logs and most *K. pneumoniae* strains by > 3 to 4 logs (Euler et al., 2023).

Additionally, some endolysins have a globular architecture and feature a cationic or amphipathic region at their C-terminus. This region binds to the negatively charged lipopolysaccharide on the bacterial surface, facilitating their passage across the OM (Figure 2). For example. The *E. coli* prophage endolysin PlyE146 is a 17 kDa protein that consists of an N-terminal N-acetylmuramidase domain and a C-terminal cationic peptide (Larpin et al., 2018). Similarly, the C-terminal region of the AcLys endolysin from *A. baumannii* AB 5075 is enriched with a stretch of arginine and lysine residues; this positively charged segment may facilitate its ability to cross the outer membrane and reach the peptidoglycan substrate (Figure 2; Sykilinda et al., 2018).

Certain Gram-negative endolysins have been identified as having a modular structure with an N-terminal CBD and C-terminal CD (Figure 2; Maciejewska et al., 2017). *P. aeruginosa* phage endolysin KZ144, OBPgp279, and EL188 feature these N-terminal CBDs and C-terminal CDs, which confer highly efficient, broad-spectrum bactericidal activity against Gram-negative strains (Briers et al., 2007; Briers et al., 2009; Walmagh et al., 2012). Similarly, *Salmonella* phage endolysins PVP-SE1gp146, SPN1S, and Gp110 also possess a large CBD and a small CD (Walmagh et al., 2012; Park et al., 2014; Rodríguez-Rubio et al., 2016). Crystal structure analyses have indicated that SPN1S possesses an enzymatic domain (Met1-Phe53 and Asp156-Ala208), alongside a small peptidoglycan-binding domain (Thr54-Gln155; Park et al., 2014).

Furthermore, protein engineering has been utilized to enhance OM permeability, thereby increasing the lytic activity of endolysins against Gram-negative bacteria. Artilysin, a trademark of Lysando AG, denotes a native lysin covalently linked to a membrane-penetrating peptide (MPP). The appended MPPs are engineered to exhibit polycationic, hydrophobic, or amphipathic properties, thereby facilitating translocation across bacterial OM (Figure 2). For instance, Art-175 is composed of an OM destabilizing peptide fused to the *P. aeruginosa* phage endolysin KZ144 (Schirmeier et al., 2018). PlyA was engineered by fusing the cecropin A peptide sequence KWKLFKKI to the *P. aeruginosa* phage endolysin OBPgp279. This modification significantly enhanced the bactericidal activity of PlyA against *A. baumannii* and *P. aeruginosa* compared with the parent OBPgp279 (Yang et al., 2015). Additionally, native endolysins can be fused with membrane-translocating domains derived from bacteriocins, which actively cross the bacterial OM to transport the endolysins into the periplasmic space, where it can reach the peptidoglycan. The surface-receptor-binding and OM translocation domains of the *P. aeruginosa* bacteriocin PyS2 were fused to the GN4 (a muramidase of *P. aeruginosa* phage PAJU2), resulting in the creation of the PyS2-GN4 lysocin. Subsequently, the antibacterial activities of GN4 and PyS2-GN4 were evaluated. Spot-test results revealed that GN4 alone exhibited no lytic activity against *P. aeruginosa*, while PyS2-GN4 demonstrated antipseudomonal activity at concentrations of ≥0.64 pmol (Heselpoth et al., 2019). Finally, a highly positively charged truncated peptide derived from phage endolysins demonstrates excellent antibacterial activity. The peptide P307, which extends from amino acids 108 to 138 in *A. baumannii* phage endolysin LysAB2, can reduce the viability of *A. baumannii* 1791, S5, and ATCC17978 strains by 3.8 logs. Furthermore, the researcher fused an eight-amino-acid segment (SQSRESQC) from the complete C-terminal region of native PlyF307 onto the C-terminus of P307, creating the variant P307_SQ-8C_, which exhibits stronger antibacterial activity than the original P307 (Thandar et al., 2016). Notably, protein engineering allows artilysin to penetrate the OM, which is also a double-edged sword and may lead to resistance. Polymyxin is a cationic peptide antibiotic that exerts its bactericidal effect by targeting the OM of Gram-negative bacteria (Thi Khanh Nhu et al., 2016). Gram-negative bacteria develop polymyxin resistance through a variety of mechanisms, including lipid A modification, LPS loss, efflux pump activation, and capsule formation (Trimble et al., 2016; Moffatt et al., 2010; Llobet et al., 2008). Therefore, we need to pay attention to the bacterial resistance caused by protein engineering. Collectively, although OM can effectively protect Gram-negative bacteria from phage endolysins, researchers continue to discover and modify these endolysins to eliminate bacteria.

Finally, we have comprehensively analyzed the structural characteristics and host range of the principal endolysins, chimeric endolysins, and artilysin derived from phages that target both Gram-positive and Gram-negative bacteria, encompassing *S. aureus*, *S. suis*, *A. baumannii*, *Klebsiella pneumoniae*, *P. aeruginosa*, and *E. coli* (Table 1). These data reveal that all examined endolysins possess a CD. Notably, enzymes targeting Gram-positive bacteria typically contain one or two CDs. This structural attribute confers upon Gram-positive bacterial endolysins a broader host range and heightened lytic efficiency. In contrast, endolysins targeting Gram-negative bacteria facilitate cell membrane penetration through the interaction with positively charged cations, thereby effectuating bacterial lysis.

**Table 1:** Characteristics of phage endolysins from important pathogens, including feature of structure and host range.

Bacteria	Phage/	Feature of structure	Host range	References
	Prophage endolysin	(N-terminal-C-terminal)		
*Staphylococcus aureus*	LysK	CHAP-Amidase-2-SH3b	Staphylococci	Yang et al. (2014)
	LSVT-1701/SAL200	CHAP-Amidase-2-SH3b	Staphylococci	Huang et al. (2021)
	LysGH15	CHAP-Amidase-2-SH3b	Staphylococci	Gu et al. (2014)
	LysP152	CHAP-SH3b	*S. aureus*	Yang et al. (2017)
	LysSAP26	CHAP	*S. aureus, A. baumannii, E. coli, K. pneumoniae, P. aeruginosa, E. faecium*	Yang et al. (2015)
	LysDZ25	CHAP-Amidase-SH3b	*S. aureus*	Vázquez et al. (2017)
	ClyS (Chimeric endolysin)	Endopeptidase-non-SH3b-like	*S. aureus*	Daniel et al. (2010)
	ClyH (Chimeric endolysin)	CHAP-non-SH3b-like	*S. aureus*	Yang et al. (2014)
	ClyF (Chimeric endolysin)	CHAP-SH3b	*S. aureus*	Yang et al. (2017)
*Streptococcus suis*	LySMP	Amidase-5-(CW_7)_2_-Glucosaminidase	*S. suis, S. aureus, S. equi s*sp. *zooepidemicus*	Wang et al. (2021), Defraine et al. (2016)
	PlySs2	CHAP-SH3b	Staphylococci, Streptococci	Gilmer et al. (2013)
	Ply30	CHAP-SH3b	*S. suis*, *S. equi* ssp. *Zooepidemicus*	Lv et al. (2015)
	Ply5218	CHAP-SH3b	*S. suis*	Shi et al. (2012)
	Ply1228	CHAP-CW_7-amidase-2	*S. suis*	Tang et al. (2015)
	Lys0859	CHAP-SH3b	Staphylococci, Streptococci	Zhang et al. (2016)
	Ply2741	CHAP-SH3b	Staphylococci, Streptococci, *E. faecalis*	Wang et al. (2025)
	ClyR (Chimeric endolysin)	CHAP-SH3b	Staphylococci, Streptococci, Enterococci	Yang et al. (2015)
	Csl2 (Chimeric endolysin)	Lysozyme-(CW_7)_2_	*S. suis*	Vázquez et al. (2017)
*Pseudomonas aeruginosa*	KZ144	N-terminal CBDs and C-terminal CDs	*P. aeruginosa*	Briers et al. (2007)
	OBPgp279	N-terminal CBDs and C-terminal CDs	*P. aeruginosa*	Walmagh et al. (2012)
	EL188	N-terminal CBDs and C-terminal CDs	*P. aeruginosa*	Briers et al. (2007)
	LysASP	lysozyme-like domain	*P. aeruginosa*	Wang et al. (2022)
	PlyPa03	Muramidase-amphipathic helix	*P. aeruginosa, Klebsiella, Enterobacter*	Yang et al. (2015)
	PlyPa91	Muramidase-amphipathic helix	*P. aeruginosa, Klebsiella, Enterobacter*	Yang et al. (2015)
	AL-3AA (Artilysin)	SMAP-29 (OM-destabilizing peptides)-LysPA26	*P. aeruginosa*	Wang et al. (2021)
*Klebsiella pneumoniae*	PlyKp104	Transglycosylase	*K. pneumoniae, P. aeruginosa, E. faecium, S. aureus, K. pneumonia, A. baumannii, P. aeruginosa, Enterobacter species*	Euler et al. (2023)
	Gp105	Transglycosylase	*E. cloacae, K. pneumoniae, P. aeruginosa, S. marcescens, Citrobacter sp., A. baumannii*	Vázquez et al. (2017)
*Acinetobacter baumannii*	LysAB2	Muramidase-amphipathic helix	*A. baumannii, S. aureus*	Cui et al. (2023)
	PlyF307	Muramidase-amphipathic helix	*A. baumannii*	Lood et al. (2015)
	LysP53	Positively charged region-peptidase	*A. baumannii, P. aeruginosa, K. pneumoniae, E. coli*	Raz et al. (2019)
	LysMK34	Muramidase-amphipathic helix	*A. baumannii, Pseudomonas aeruginosa*	Wang et al. (2021)
	Art-175 (Artilysin)	SMAP-29-KZ144	*A. baumannii*	Defraine et al. (2016)
*Escherichia coli*	PlyE146	Acetylmuramidase-cationic peptide	*E. coli, P. aeruginosa, A. baumannii*	Larpin et al. (2018)
	PlyEc2	Acetylmuramidase-cationic peptide	*E. coli, Salmonella, Shigella, Acinetobacter, Pseudomonas*	Ramesh et al. (2022)
	Lysep3	Lysozyme	*E. coli*	Lv et al. (2015)

## 3 Therapeutic efficacy of phage endolysins

Over the past two decades, extensive research has investigated the antibacterial activity of endolysins through *in vitro* assays and animal infection/colonization models, yielding significantly promising results (Nelson et al., 2012; Pastagia et al., 2013; Roach and Donovan, 2015; Haddad Kashani et al., 2018). In this section, we review the use of phage endolysins in animal infection models and human clinical trials before their formal introduction for treating bacterial infections in clinical practice.

### 3.1 Evaluation of phage endolysins in animal models of bacterial infections

Numerous endolysins have been investigated and demonstrated efficacy against a variety of lethal pathogens. Infections caused by drug-resistant Gram-positive bacteria, such as staphylococci, represent a serious worldwide public-health problem, significantly increasing morbidity, mortality, and healthcare costs (Golban et al., 2025). *S. aureus*, an important species of the *Staphylococcus* genus, is a prevalent opportunistic pathogen that colonizes the nasal mucosa of approximately 20–40% of individuals, playing a crucial role in the transmission of both hospital-acquired and community-acquired infections. *S. aureus* has developed high-level resistance to antibiotics, including penicillin, azithromycin, and cefoxitin (Congdon et al., 2023). Phage endolysins have consistently demonstrated efficacy against staphylococcal infections in both *in vitro* and *in vivo* studies.

*S. aureus* phage lysin LysGH15, *Streptococcus suis* prophage lysin PlySs2 (also known as CF-301), and staphylococcal phage lysin LSVT-1701 (previously known as SAL200) have been widely investigated for their potential use in treating staphylococcal infections (Gu et al., 2011). *S. aureus* phage endolysin LysGH15, *Streptococcus suis* prophage endolysin PlySs2 (also known as CF-301), and staphylococcal phage endolysin LSVT-1701 (previously known as SAL200) have been widely investigated for their potential use in treating staphylococcal infections (Gu et al., 2011). LysGH15 is a endolysin from the *S. aureus* phage GH15 and exhibits a broad host range against methicillin-resistant *S. aureus* (MRSA) and methicillin-susceptible *S. aureus* (MSSA) strains isolated from patients at the First Hospital of Jilin University. Notably, LysGH15 does not exhibit lytic activity against S*treptococcus*, *B. subtilis*, *Salmonella enteritidis*, *K. pneumoniae*, and *E. coli*. Notably, LysGH15 is the first *Staphylococcus* phage endolysin whose protein structure has been resolved (Gu et al., 2014). In the mouse bacteremia model, LysGH15 treatment with 5, 10, and 50 μg/mouse increased the survival rate of mice infected with lethal doses of MRSA YB57 to 0, 40, and 100%, respectively (Gu et al., 2011). In the mouse model of *S. aureus*-induced pneumonia, the mice infected with 2 × MLD (1 × 10^8^ CFU) of MRSA USA300 strain received one of three treatments 1 h later (n = 10 per group): Intranasal administration of LysGH15 alone (60 μg per mouse), subcutaneous injection of apigenin alone (500 μg), or a combination of both agents. The results indicated that the survival rate of mice treated with LysGH15 alone and apigenin alone was 80% (8/10) and 0% (0/10), respectively. However, the combination of LysGH15 and apigenin resulted in a 100% survival rate (10/10). Furthermore, the lungs of mice receiving the combined treatment of LysGH15 and apigenin exhibited no observed inflammation or other pathological alterations, and the concentrations of tumor necrosis factor-*α* (TNF-α), IL-1β, and IL-6 were the most comparable to those detected in health mice (Xia et al., 2016). In the rabbit model of *S. aureus*-induced pneumonia, a single intranasal dose of 300 μg per rabbit increased the survival rate to 60%. The bacterial load in the lung tissue of rabbits treated with LysGH15 was 7 × 10^4^ CFU/g, which was significantly lower than that observed in rabbits receiving PBS (7.76 × 10^6^ CFU/g) or linezolid (6.38 × 10^5^ CFU/g). Moreover, the combination of LysGH15 and apigenin demonstrates its potential for treating MRSA-induced skin infections. Cheng et al. (2018) formulated an LAA ointment by mixing freeze-dried LysGH15 (containing 50 μg LysGH13 per 0.1 g of ointment) and apigenin (500 μg per 0.1 g of ointment) with Aquaphor. This 0.1 g ointment reduced wound areas by 65.46% 5 days after infection and decreased the bacterial burden in skin infected with USA300 cultures by 3.3 CFU/mg at 18 h post-treatment compared with PBS treatment.

*S. suis* prophage endolysin PlySs2 demonstrates a broad host range, effectively targeting *Streptococcus* and *Staphylococcus* (Gilmer et al., 2013). In the mouse bacteremia model of mixed MRSA MW2 strain and *Streptococcus pyogenes* MGAS 5005 strain infection, a single 2-mg dose of PlySs2 protected 92% of the mice (22 out of 24). Following treatment with 1 mg of PlySs2 per mouse, the survival rate reached 89% (16 out of 18) for mice challenged with the MW2 strain, and 94% (15 out of 16) for those challenged with the MGAS 5005 strain (Gilmer et al., 2013). As a *S. suis* phage endolysin, PlySs2 displays lytic activity against multiple serotypes of *S. suis* strains. In the *S. suis* nasal mucosal colonization model, mice were intranasally administered 1 × 10^7^ CFU of the 7,997 strain in each nostril and treated with 100 μg/mouse PlySs2 and a combination of 50 μg/mouse PlySs2 and 50 μg/mouse gentamicin after 24 h of infection by nasal injection. Following treatment, the burden of *S. suis* strain 7,997 in the nasal mucosa treated intranasally with PlySs2 and the combination was reduced by > 4 logs and > 5 logs, respectively (Gilmer et al., 2017). Within a rat model of MRSA-induced acute osteomyelitis, the model was built by injecting 10^7^ CFU of the MRSA IDRL-6169 strain into the tibia. Intraperitoneal injection of daptomycin (60 mg/kg), CF-301 (40 mg/kg), and a combination of both reduced the number of bacteria in bone, yielding reductions of 4.09 (±0.37), 4.65 (±0.65), and 3.57 (±0.48) log_10_ CFU/g, respectively (Karau et al., 2019). This study represents a pioneering effort to employ phage endolysin treatment for osteomyelitis. In the rabbit model of osteomyelitis with bone-implanted screws colonized with MRSA, CF-296, an engineered lysin of CF-301, the combination of CF-296 and daptomycin displayed the greater efficacy than either CF-296 alone (*p* = 0.0040) or daptomycin alone (*p* = 0.0098) when evaluated using bone cultures (Karau et al., 2022). Simultaneously, in the mouse model of neutropenic thigh infection, the number of viable *S. aureus* (1 MSSA, 7 MRSA) clinical isolates in the thigh treated subcutaneously with CF-301 and a combination of was reduced by 0.77 ± 0.98 to 1.20 ± 0.59 logs at 24 h post-infection, respectively (Asempa et al., 2020). Within the rabbit model of MRSA endocarditis, a combination of CF-301 (11 mg/kg) and daptomycin (4 mg/kg) resulted in a significant reduction of > 8 log_10_ CFU/g (*p* < 0.0001). However, daptomycin alone only caused a significant reduction of 3 log_10_ CFU/g (*p* < 0.0001; Shah et al., 2020). Within the lethal mouse model of *S. aureus* pneumonia infection, a single daily intravenous dose of exebacase CF-301 at 5 mg/kg for three consecutive days achieved a 50% survival rate, whereas the vehicle-treated control group indicated 0% survival. When CF-301 (5 mg/kg/day for 3 days) is combined with antibiotics daptomycin (50 mg/kg/day for 3 days), the survival rate of the mice increased to 70% (Swift et al., 2021).

Staphylococcal phage endolysin SAL200 (containing phage SAP1 endolysin SAL-1) possesses an excellent antibacterial activity against 425 clinical isolates (including 336 MRSA and 1 vancomycin-intermediate *S. aureus*) and 415 clinical isolates (including 315 *S. aureus* and 100 coagulase-negative staphylococci) from 2002 to 2019 (Huang et al., 2021; Jun et al., 2013). In the mouse model of bacteremia, the mice were infected with the 1 × 10^8^ CFU/mouse *S. aureus* isolate SA2 by intravenous injection and treated with 12.5 and 25 mg/kg of SAL200 at 1, 25, and 49 h post-infection by intravenous injection. The survival rate of mice treated with 12.5 and 25 mg/kg of SAL200 reaches 93.3% (14/15) and 100% (15/15) at 5 days post-infection, respectively. Meanwhile, the number of bacteria in the serum and spleen was decreased by > 5 logs and > 7 logs, respectively (Jun et al., 2013). In the lethal mouse model of *S. aureus*-induced pneumonia, the survival rates of mice treated with SAL200 (0.3 mg/mouse) and PBS (30 μL/mouse) at 2 h post-infection were 95–100% (n = 20) and 6.7–40% (n = 20). Notably, although SAL200 significantly reduced the number of bacteria in serum and lung tissue compared to PBS-treated groups, no significant decreases in the cytokine levels of IL-2, IL-4, IL-5, IL-10, IL-12, IFN-*γ*, and TNF-*α* were observed in the serum of SAL200-treated groups compared with the control group until 10 h post-infection (Bae et al., 2019). The previous studies indicated that phage endolysins can reduce the cytokine levels in the lung in the mouse model of pneumonia (Xia et al., 2016; Doehn et al., 2013). Collectively, SAL200 holds potential as an innovative antibacterial agent against *Staphylococcus* infections.

Currently, there are extensive reports on the phage endolysins and chimeric endolysins that can treat bacterial infection in different animal models. For instance, *Aerococcus viridans* phage endolysin AVPL, *A. baumannii* prophage endolysin PlyF307, *S. suis* prophage endolysin Ply2741 and Lys0859, and *Streptococcus dysgalactiae* prophage endolysin PlySK1249 can treat the Gram-positive and Gram-negative bacteria-induced mouse bacteremia (Li et al., 2023; Oechslin et al., 2013; Xi et al., 2023; Lood et al., 2015; Wang et al., 2025). The MRSA-induced mouse skin and wound infection can be effectively treated by chimeric endolysins ClyQ and ClyS (Duan et al., 2023; Pastagia et al., 2011). Moreover, *K. pneumoniae* prophage endolysin PlyKp104 and *Acinetobacter baumannii* phage endolysin LysP53 possess the potential of treating the Gram-negative bacteria-induced mouse pneumonia (Euler et al., 2023).

In summary, phage endolysins are emerging as novel antibacterial candidates with potent bactericidal activity, high target specificity, and minimal resistance development. Multiple endolysins have entered clinical evaluation, particularly for infections caused by multidrug-resistant Gram-positive bacteria, with promising safety and efficacy. Although challenges such as *in vivo* stability, delivery, and activity against Gram-negative pathogens persist, continuous engineering advances are accelerating their clinical translation (Cui et al., 2025). Overall, phage endolysins represent a promising strategy to address the global threat of antimicrobial resistance.

### 3.2 Evaluation of phage endolysins in human clinical trials

Phage endolysins have undergone multiple clinical studies, and maintaining their activity stability in serum is a prerequisite for clinical trials. Conversely, human serum concentrations of 12% or higher suppressed PlyKp104 activity. However, at just 6% serum concentration, the endolysin decreased viable *K. pneumoniae* and *P. aeruginosa* by more than 2.5-log and 5-log, respectively (Euler et al., 2023). The *in vitro* experiments demonstrated that the bacteriolytic activity of *S. suis* prophage endolysin Ply5218 progressively decreased with longer incubation times in pig serum (Wang et al., 2019). Thandar et al. (2016) evaluated the peptide activities in human blood plasma and found that, at a concentration of 100 μg/mL, neither P307 nor P307SQ-8C exhibited any activity in undiluted plasma. Conversely, the results of minimum inhibitory concentrations (MICs), checkerboard synergy, and time-kill assays indicated that CF-301 demonstrated a significantly higher potency, approximately 32- to 100-fold greater in human blood compared with cation-adjusted Mueller-Hinton broth. Chiara *et al*. reported that this increased efficacy is linked to the systemic bactericidal action of CF-301, along with two human blood factors: Human serum lysozyme and human serum albumin (Indiani et al., 2019). These findings indicate that serum exerts a highly specific, endolysin-dependent effect on enzymatic activity, rather than a uniform or consistent regulatory pattern across all phage lysins tested. Researchers need to focus on enhancing the activity and stability of phage endolysins in serum for improved effectiveness.

This section summarizes the current clinical research progress regarding phage endolysins. The Phase I clinical trial: Jun et al. (2017) assessed the safety and tolerance of single, ascending intravenous doses of SAL200 in 34 healthy Korean male volunteers. These participants were randomly assigned to receive either SAL200 or a placebo across five dose cohorts (0.1, 0.3, 1, 3, and 10 mg/kg body weight). This trial represents the first-in-human, Phase I study of an intravenously delivered drug derived from a phage endolysin (Clinicaltrials.gov identifier: NCT01855048). SAL200 was well tolerated, with no adverse events or laboratory abnormalities observed after administering single doses of up to 80 mg/kg per day or repeated doses of up to 40 mg/kg per day (Jun et al., 2016). In another phase I study of SAL200, LSVT-1701/SAL200 was administered intravenously as a 6 mg/kg single dose of LSVT-1701 or placebo, followed by 1.5, 3, and 4.5 mg/kg doses twice daily for 4 days. The LSVT-1701 group was safe and well-tolerated. Most treatment-emergent adverse events (TEAEs) were mild (97%); there were no severe or serious adverse events, no deaths, and no withdrawals due to AEs. In total, 15 of the 32 participants (47%) experienced the TEAEs (Clinicaltrials.gov identifier: NCT03446053; Wire et al., 2022). Additionally, Moorthy et al. (2022) described the first infant to receive phage endolysin, finding that exebacase CF301 may be safe and efficacious in children. In summary, phage endolysins demonstrate favorable safety and tolerance in the body.

The Phase II clinical trial: In one study, 121 patients with *S. aureus* bloodstream infection (BSI) or endocarditis were randomly assigned to receive either a single dose of CF301 (45 completed patients) or placebo (48 completed patients), along with SOC antibiotic therapy (vancomycin and *β*-lactams). The primary efficacy endpoint was the clinical outcome, defined as the responder rate assessed at day 14 (Clinicaltrials.gov identifier: NCT03163446). At day 14, the proportion of clinical responders in the group treated with both endolysins and antibiotics was 70.4%, compared to 60.0% in the antibiotic-only group. The 30-day all-cause mortality rate was 9.7% in the exebacase + antibiotics group, compared to 12.8% in the antibiotics-only group. This study provides proof-of-concept evidence that phage endolysin agents can serve as potential therapeutics, supporting the need for a confirmatory trial focusing on endolysin for the treatment of MRSA-induced BSI (Fowler et al., 2020). In the phase II clinical study of SAL200, a randomized, double-blind, placebo-controlled, multicenter trial was conducted to evaluate the safety and explore the efficacy of SAL200 in patients with persistent *S. aureus* bacteremia lasting more than 48 h despite treatment with susceptible antibiotics. This study was conducted to assess the safety and explore the efficacy of a single intravenous dose of N-Rephasin® SAL200 (3 mg/kg) alongside conventional standard therapy. The results revealed that placebo and SAL200 groups exhibited similar rates of treatment-emergent adverse events (Phase IIa Clinical Study of N-Rephasin® SAL200, 2021). In conclusion, the combination of phage endolysins and antibiotics can effectively treat bacterial infections.

The Phase III clinical trial: Currently, CF301 is the only phage endolysin that has successfully completed Phase III clinical trials (Clinicaltrials.gov identifier: NCT04160468). The primary objectives were to assess the safety and tolerability of CF301 combined with antibiotics and to determine whether this combination achieved a superior clinical response rate at day 14 compared with antibiotics alone. The secondary objectives included evaluating whether CF301 plus antibiotics produced a higher clinical response rate at day 14 than antibiotics alone. A total of 259 patients with *S. aureus*-positive blood cultures and BSIs were enrolled, with samples collected within 72 h before randomization. Patients were randomly assigned in a 2:1 ratio to receive either CF301 or placebo, in addition to either daptomycin or vancomycin. The rate of TEAEs was similar in CF301 + antibiotic and antibiotic alone groups. However, the clinical response rate of day 14 among the MRSA cohort (*n* = 97) revealed that 50.0% of patients receiving CF301 combined with antibiotics (32/64) responded positively, compared to a response rate of 60.6% in the antibiotics alone group (20/33; Fowler et al., 2024). Prior research indicates that endolysins possess the capacity to elicit an immune response *in vivo*. Hyperimmune serum significantly reduces the in-vitro activity of Cpl-1, although it does not completely inactivate the enzyme (Gondil et al., 2020). In addition, an expanding body of evidence demonstrates that phages are capable of interacting with mammals, specifically humans (Bodner et al., 2021; Hamdi et al., 2025). Phages display pharmacokinetic characteristics that differ from those of small-molecule antimicrobial agents, owing to their substantial size, elevated protein composition, and inherent ability to replicate autonomously (Zou et al., 2023; Dąbrowska and Abedon, 2019). These factors may collectively account for the failure of Phase III clinical trials. Although CF301 did not demonstrate an enhanced clinical response, the trial provides valuable insights for designing future studies aimed at treating *S. aureus* bacteremia and endocarditis.

**Figure 3:**
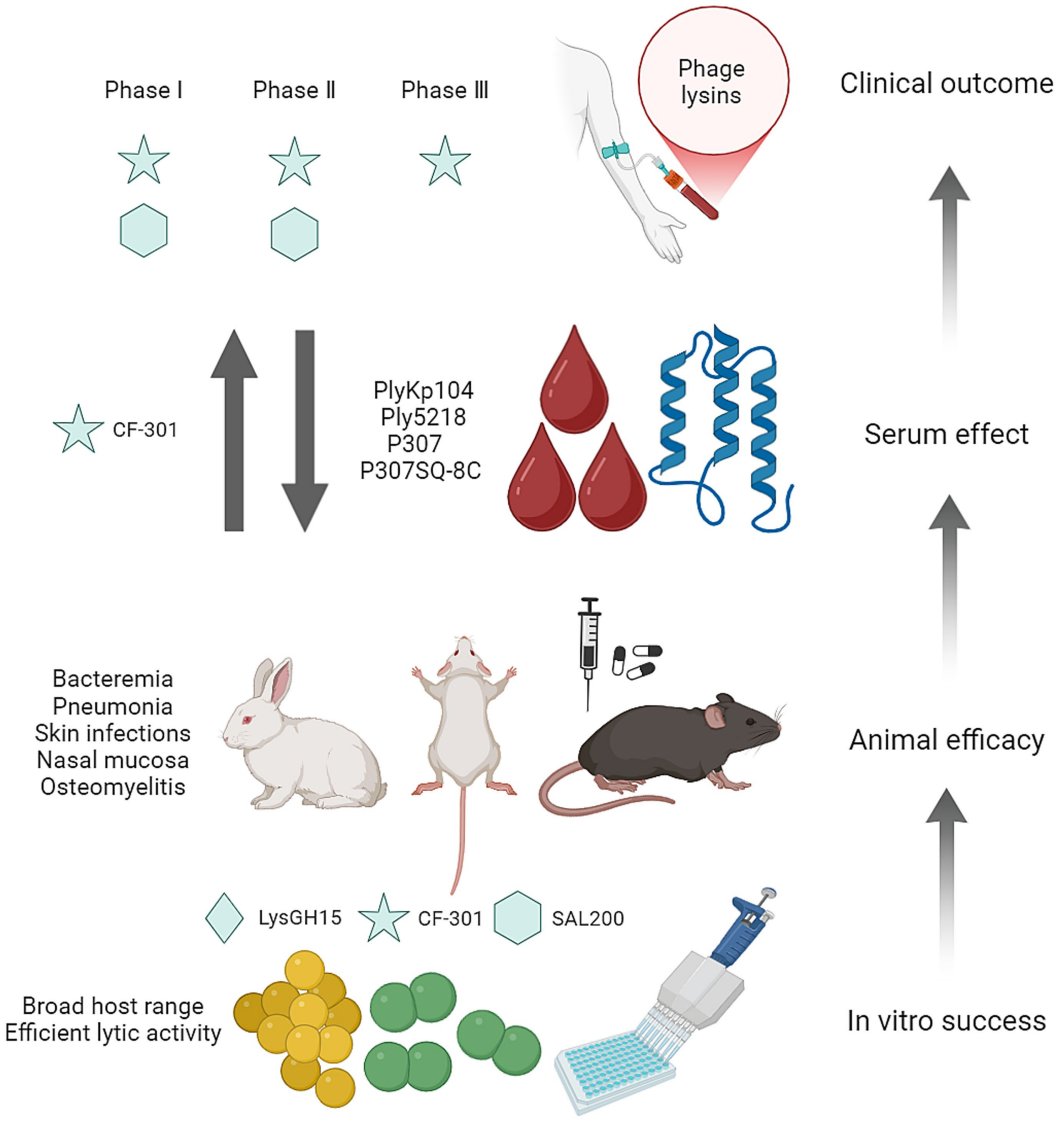
Therapeutic efficacy of phage endolysins. These valuable *in vitro* and *in vivo* data underpin the potential clinical applications of phage lysins.

In conclusion, phage endolysins possess the broad host range and excellent antibcterial activity against different bacteria, especially *Staphylococcus* and *Streptococcus in vitro*. Subsequently, phage endolysins LysGH15, CF-301, and SAL200 exhibit the bactericidal activity in the animal models of bacteremia, pneumonia, skin infections, nasal mucosa infections, and osteomyelitis. However, the effect of serum on endolysin activity is enzyme-specific; it inhibits the activity of endolysins PlyKp104, Ply5218, P307, and P307SQ-8C, while enhancing the activity of CF-301. This dichotomous effect warrants careful consideration during subsequent enzyme modification and application. Finally, both CF-301 and SAL200 have successfully completed Phase I and Phase II clinical trials, and CF-301 have conducted Phase III clinical trials (Figure 3). These valuable *in vitro* and *in vivo* data underpin the potential clinical applications of phage lysins.

## 4 Resistance of phage endolysins

Current research indicates that inducing bacterial resistance to phage endolysins is challenging (Duan et al., 2023; Daniel et al., 2010; Ho et al., 2022; Yang et al., 2019; Loeffler et al., 2001). Several reasons contribute to this difficulty, for instance, PG is the targeted site of phage endolysins and highly conserved and virtually immutable, with no resistance reported after exogenous application of endolysins (Gerstmans et al., 2018). Additionally, major resistance mechanisms against antimicrobials are typically located inside the cell, while the extracellular application of endolysins and the presence of target peptidoglycan on the outer cell surface limit the potential for resistance development (Spratt, 1994). In summary, there have been no reported cases of bacterial resistance induced by phage endolysins, and the risks associated with long-term and extensive use of these endolysins are still unclear. However, numerous bacterial species, such as *S. aureus*, *S. suis*, and *Bacillus subtilis*, have evolved lysozyme resistance by modifying the structure of their peptidoglycan (Jones et al., 2021; Ho et al., 2011; Bera et al., 2006; Herbert et al., 2007; Guariglia-Oropeza and Helmann, 2011; Wichgers Schreur et al., 2012). Previous researches reported that the enzyme serine hydroxymethyltransferase, WTAs and *femA* gene significantly contributes to the lysostaphin resistance of *S. aureus* (Climo et al., 2001; Batool et al., 2020; Wu et al., 2019). Consequently, proactive strategies should be implemented to anticipate and mitigate the emergence of resistance to phage endolysins during its large-scale deployment. This section speculates on the potential emergence of resistance to phage endolysins based on current knowledge and logically infers the underlying molecular mechanisms that might be involved.

### 4.1 Immediate stress responses

For any organism, the ability to anticipate and proactively counter potential threats is widely considered to confer a selective survival advantage. Our first objective is to investigate how bacteria perceive and respond to danger signals. On the one hand, quorum sensing (QS) is a mechanism of chemical communication between bacterial cells, which relies on the production, detection, and subsequent response to extracellular signaling molecules known as autoinducers (Mukherjee and Bassler, 2019). QS is recognized as a cell-density-dependent signaling phenomenon that regulates various essential biological processes, particularly virulence and biofilm formation (Mizan et al., 2016; Yuan et al., 2018). Conversely, two-component systems (TCSs) are pivotal mechanisms through which bacteria sense and respond to environmental changes. These systems consist of a membrane-anchored sensor histidine kinase (HK) and a cytoplasmic response regulator (RR), enabling bacteria to detect extracellular signals, metabolic by products, temperature variations, pH changes, and various danger signals (West and Stock, 2001; Trouillon et al., 2021; Yu et al., 2022; Daddaoua et al., 2014; Chakraborty et al., 2010; Loh et al., 2021; Tierney and Rather, 2019). In Gram-positive bacteria, oligopeptides often serve as autoinducers, and their corresponding receptors are transmembrane HKs within TCSs (Waters and Bassler, 2005). For instance, *S. aureus* possesses two QS systems, Agr-QS and AI-2 QS, both of which serve as pivotal regulators of its pathogenicity and antibiotic resistance (Fan et al., 2022; Kim et al., 2017; Ma et al., 2017). Agr-QS consists of the *agrBDCA* operon and *RNA III* gene in *S. aureus* (Li et al., 2025). At high cell density, the *agrBDCA* operon is activated and produces autoinducing peptides (AIPs). TCSs (AgrC/AgrA) then sense these AIPs, leading to regulation of gene expression (Painter et al., 2014). In natural environments, the likelihood of phage infection escalates with increasing bacterial population density. At high cell density, bacteria utilize QS to control their responses to phage threats. Activation of the Agr-QS system enhances phage adsorption by down-regulating tarM expression, which reduces *α*-GlcNAc glycosylation of WTA and facilitates phage infection (Yang et al., 2023). Yang et al. (2023) also found that higher cell densities diminish the expression of cas genes in *S. aureus*, weakening CRISPR-Cas-mediated antiphage immunity and increasing bacterial susceptibility to phage infection. Notably, QS RR AgrA represses the expression of other regulators, such as RR *SarA* and *ArcR*, thereby inhibiting CRISPR1 and *cas* and ultimately impairing the efficacy of the anti-phage system (Li et al., 2025). Conversely, at high cell density, antiphage system is upregulated by QS in *P. aeruginosa*, *Aliivibrio wodanis*, and *Serratia* (Broniewski et al., 2021; Høyland-Kroghsbo et al., 2017; Maharajan et al., 2022; Patterson et al., 2016).

**Figure 4:**
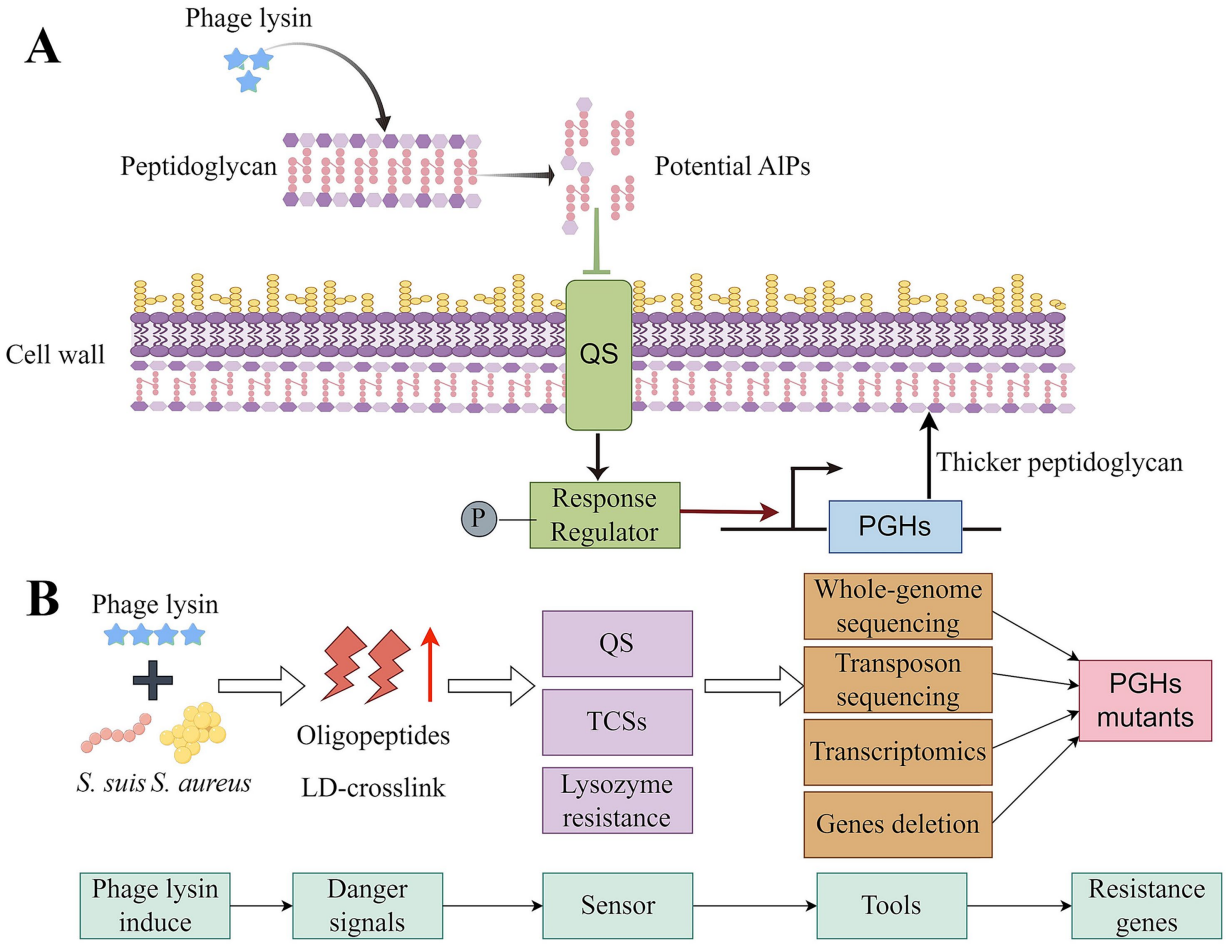
The potential mechanisms of resistance to phage endolysins. **(A)** Bacteria develop resistance to phage endolysins through QS. Peptides or glycopeptides produced by phage endolysin-derived lysate may serve as autoinducers of QS to stimulate live bacteria that have not been lysed by phage endolysins. **(B)** Possible methods for revealing the mechanism of phage endolysin resistance. The primary task is to enable researchers to induce bacterial resistance in the lab by treating with endolysins at varying concentrations. Oligopeptides and LD-Crosslink produced by bacterial lysis may be danger signals that induce resistance. Researchers may explore the effects of changes in bacterial phage endolysin resistance through combined QS and TCS systems, or TCSs alone. The Tn mutant libraries of bacteria and the MIC results from these mutants can be used to screen for key genes related to phage endolysin resistance.

Based on the mechanism of action of phage endolysins, peptides or glycopeptides produced by phage endolysin-derived lysate may serve as autoinducers of QS to stimulate live bacteria that have not been lysed by phage endolysins. This indicates that phage endolysins might alter the density of targeted bacteria, which would be expected to activate QS-related TCS and putatively regulate the expression of associated genes. When phage endolysins are added to bacteria, we can monitor its impact on the expression of genes involved in peptidoglycan or cell wall synthesis using transcriptomics (Figure 4A). To our knowledge, established methods exist for gene deletion and mutation in *S. aureus* and *S. suis* (Li et al., 2025; Kato and Sugai, 2011; Takamatsu et al., 2001), allowing for validating related genes affecting phage endolysin resistance through gene deletion and MICs. Finally, regulatory factors (TCSs) can be screened using DNA pull-down techniques. Researchers may explore the effects of changes in bacterial phage endolysin resistance through combined QS and TCS systems, or TCSs alone (Figure 4B; Li et al., 2025; Chao et al., 2016). Furthermore, Sanika Vaidya et al. (2025) reported that peptidoglycan fragments act as a universal danger signal that can induce biofilm formation in both Gram-negative and Gram-positive bacteria, thereby helping these microbes evade phage predation. Phage endolysins are known to rapidly lyse peptidoglycan, leading to extracellular accumulation of peptidoglycan fragments that may function as potent danger signals for neighboring intact bacteria. These observations lead us to hypothesize that peptidoglycan fragments could contribute to bacterial evasion from phage endolysin-mediated degradation.

### 4.2 Evolutionary resistance pathways

It has been observed that the concentration of phage endolysin typically ranges from 1/32 to 4 × MIC (Pastagia et al., 2011; Yang et al., 2019). In clinical trials, the concentration of phage endolysins can reach 5 mg/kg (Fowler et al., 2024). Consequently, researchers should perform the experiment with various concentrations of endolysins in the laboratory, from low to high, to induce bacterial resistance (Figure 4B; Suzuki et al., 2014). Notably, bacterial cells can express hundreds of genes related to antibiotic resistance during laboratory evolution (Suzuki et al., 2014). Researchers can employ machine learning and statistical models to identify potential genes associated with phage endolysin resistance through phenotypic testing (Suzuki et al., 2014; Su et al., 2019). When coupled with high-throughput sequencing, transposon sequencing offers an efficient approach for genome-wide identification of essential genes, including virulence determinants and AMR genes (Chao et al., 2016). Researchers can construct the Tn mutant libraries of *S. aureus* or *S. suis*, and the MIC results from these mutants can be used to screen for key genes related to phage endolysin resistance (Fan et al., 2022; Lo et al., 2023).

### 4.3 Resistance genes and structural adaptation

To investigate genes involved in phage endolysin resistance, the effect of CD and CBD was evaluated on the antibacterial activity of phage endolysins. The peptidoglycan layer is the target site for CD, and thus, genes involved in peptidoglycan and cell wall biosynthesis warrant focused investigation. The coordinated action of peptidoglycan synthases and hydrolases is crucial for cellular growth and division (Turner et al., 2014). Peptidoglycan hydrolases (PGHs) or autolysins hydrolyze covalent bonds within the existing peptidoglycan structure, facilitating the incorporation of newly synthesized material, which is essential for bacterial growth, division, and separation (Alvarez et al., 2024; Leonard et al., 2023). The results of Joshua *et al*. revealed that the absence of PGH SagB is associated with thickening of the *S. aureus* cell wall (Sutton et al., 2021). This suggests that the mutant PGH mutants require more phage endolysins to lyse their cell walls and may represent key genes for phage endolysin resistance. The presence of LD-crosslink can strengthen the cell wall and serve as inhibitors of the activity of autolysin lytic LTs (Alvarez et al., 2024; Magnet et al., 2008; Mainardi et al., 2005). We hypothesize that lysates derived from phage endolysins generate LD-crosslinks, which suppress the activity of PGHs. Even when phage endolysins continuously induces bacterial cells, it can lead to mutations in PGH genes. Consequently, the bacterial cell wall becomes thicker, leading to the emergence of resistance to phage endolysins (Figure 4B).

Lysozyme catalyzes the hydrolysis of *β*-1,4-glycosidic linkages (Yang and Yan, 2025), and the molecular mechanisms by which bacteria acquire resistance to lysozyme are well-documented. Understanding these mechanisms of lysozyme resistance can help explore the pathway to phage endolysin resistance. MurNAc *O*-acetylation is prevalent among Gram-positive bacteria and is often associated with enhanced resistance to lysozyme and endogenous autolysins. Notably, *O*-acetyltransferase (*OatA*) and *OatB* genes are responsible for *O*-acetylation of MurNAc and GlcNAc, which causes the resistance of lysozyme (Jones et al., 2021; Bernard et al., 2011). Additionally, evidence suggests that the activity of phage endolysins can be influenced by modifications to the peptidoglycan layer (Grishin et al., 2020). Consequently, it is essential to determine whether the genes linked to lysozyme resistance and peptidoglycan modifications are also responsible for phage lytic enzyme resistance (Figure 4B).

## 5 Future prospects

Recent advances in research have introduced novel β-lactamase inhibitor (BLI) combinations into clinical practice, including ceftolozane/tazobactam, ceftazidime/avibactam, meropenem/vaborbactam, and imipenem/cilastatin/relebactam (Barbier et al., 2023). The synergistic combination of targeted inhibitors with conventional antibiotics emerges as a promising therapeutic avenue for combating infections caused by multidrug-resistant pathogens. Given the limited emergence of resistance to phage endolysins, future endolysins development must incorporate resistance surveillance early in the preclinical pipeline. β-Lactamases catalyze the hydrolysis of β-lactam antibiotics, thereby constituting the principal mechanism underlying bacterial resistance to these agents (Agarwal et al., 2023). Therefore, we need to reveal the mechanism of phage endolysin resistance and and mitigate the risk of resistance emergence in clinical settings.

On the one hand, QS inhibitors are compounds that interfere with bacterial QS systems, thereby attenuating virulence. An increasing body of research suggests that QS inhibitors have been employed in the development of therapeutics for bacterial infections (Zhang et al., 2024; Xiang et al., 2021). We reasonably hypothesize that QS inhibitors can effectively suppress the phage endolysin-mediated alterations in AIPs induced by bacterial detection. On the other hand, by elucidating the mechanism through which the regulatory system controls the resistance genes and utilizing machine learning prediction to identify potential inhibitors of resistance. Finally, the combination of phage endolysins and antibiotics demonstrates synergistic bactericidal activity (Swift et al., 2021; Watson et al., 2020), offering a promising strategy against drug resistance.
